# The Expression of Porcine *Prdx6* Gene Is Up-Regulated by C/EBPβ and CREB

**DOI:** 10.1371/journal.pone.0144851

**Published:** 2015-12-14

**Authors:** Xinyu Wu, Panlong Ji, Liang Zhang, Guowei Bu, Hao Gu, Xiaojing Wang, Yuanzhu Xiong, Bo Zuo

**Affiliations:** 1 Key Laboratory of Swine Genetics and Breeding, Ministry of Agriculture and Key Lab of Agricultural Animal Genetics and Breeding, Ministry of Education, College of Animal Science and Veterinary Medicine, Huazhong Agricultural University, Wuhan, 430070, P.R. China; 2 Department of Basic Veterinary Physiology and Biochemistry Laboratory, Huazhong Agricultural University, Wuhan, 430070, PR China; Wageningen UR Livestock Research, NETHERLANDS

## Abstract

Peroxiredoxin6 (Prdx6) is one of the peroxiredoxin (Prdxs) family members that play an important role in maintaining cell homeostasis. Our previous studies demonstrated that *Prdx6* was significantly associated with pig meat quality, especially meat tenderness. However, the transcriptional regulation of porcine *Prdx6* remains unclear. In this study, we determined the transcription start site (TSS) of porcine *Prdx6* gene by 5′ rapid-amplification of cDNA ends (5′ RACE). Several regulatory elements including CCAAT/enhancer-binding proteinβ (C/EBPβ), Myogenic Differentiation (MyoD), cAMP response element binding protein (CREB), stimulating protein1 (Sp1) and heat shock factor (HSF) binding sites were found by computational analyses together with luciferase reporter system. Overexpression and RNA interference experiments showed that C/EBPβ or CREB could up-regulate the expression of porcine *Prdx6* gene at both mRNA and protein level. Electrophoretic mobility shift assays (EMSA) and chromatin immunoprecipitation assays (ChIP) confirmed that C/EBPβ and CREB could interact with *Prdx6* promoter. Immuoprecipitation results also showed that C/EBPβ could interact with Prdx6 *in vivo*. Taken together, our findings identified C/EBPβ and CREB as the important regulators of porcine *Prdx6* gene expression, and offered clues for further investigation of *Prdx6* gene function.

## Introduction

Peroxiredoxins are a widely distributed superfamily of peroxidases with high antioxidant activity and have been received much more attention in recent years [1, 2]. Six peroxiredoxin family numbers (Prdx1-6) have been identified in mammals and classified as 1-cys, 2-cys based on the number of conserved cystines by which thiol groups are formed as catalytic center to degrade H_2_O_2_ and other peroxides [3, 4]. Prdx1-5 belongs to 2-cys peroxiredoxins and utilizes thioredoxin as a reductant. Unlike other peroxiredoxins, Prdx6 is the only 1-cysteine peroxiredoxin, and uses glutathione (GSH) rather than thioredoxin as the electron donor [5–8].

Prdx6 is widely distributed in all major organs with high expression levels in lung, kidney, liver, eye and testes [9–12]. It is a bifunctional enzyme with both GSH peroxidase and acidic Ca^2+^ independent phospholipase A_2_ activities that could participate in many physiological processes [7]. With the phospholipase A_2_ activity, Prdx6 can regulate phospholipid metabolism. And the GSH peroxidase activity allows Prdx6 to protect cells against oxidative stress by removing reactive oxygen species (ROSs) and limiting ROS levels. ROS are generated in cells during several normal physiologic processes and will be immediately removed by antioxidants like Prdx6. When the equilibrium status between the production and elimination of reactive oxygen species is broken, oxidative stress occur [13]. Excess ROS can result in irreversible oxidative damage to cellular lipids, DNA and proteins, and thereby caused many human diseases [13, 14]. Prdx6 has been reported to implicate in development and progression of several human diseases, like Alzheimer [15], Parkinson dementia [16], diabetes [17], cancer [18–21], cataractogenesis [22]. The formation mechanism of those diseases mostly related to oxidative stress. By analyzing the *Prdx6* transcription regulation, many researchers have identified the mainly redox-active regulator of this gene in human, rat or mouse, like Nrf2 [23–25], NF-κB [26, 27], Sp1 [28], AP1 [18], HSF1 [29].

In animal production, research has shown that the Prdx6 might be a potential protein marker for meat tenderness in bovine biopsies and samples collected shortly after slaughter [30]. Meat tenderness is an important meat eating quality and has achieved much attention in modern animal production. Studies in our laboratory found that *Prdx6* gene was differentially expressed in longissimus dorsi between different pig breeds (data unpublished). Besides, two SNPs (417bp C/T; 423bp A/G) were detected in the fourth exon of porcine *Prdx6* gene, and were significantly associated with meat quality, especially meat tenderness [31]. The above findings indicated the new function of Prdx6 expression in pig meat quality traits. However, how the expression of *Prdx6* gene is regulated is still unclear in pigs until now. In this study, the porcine *Prdx6* promoter region and its transcription start site were identified and the possible transcription factors for the *Prdx6* gene regulation were also investigated. We found that the transcription factor C/EBPβ and CREB could bind to their binding sites in *Prdx6* promoter and regulate the expression of target genes. This study provided the insight into the basal regulation mechanisms of porcine *Prdx6* gene, and the possible signaling pathway for the participation of *Prdx6* gene in the adipogenesis and myogenesis.

## Material and Methods

### Animals, tissues and cell lines

The animal experimental procedures were approved by the Huazhong Agricultural University Institutional Animal Care and Use Committee, Wuhan, China. All pigs used in this study were from the Pig Farm of Huazhong agricultural university. The pigs were slaughtered after low voltage electrical stunning. Ten tissues including kidney, small intestine, stomach, longissimus dorsi, fat, brain, heart, liver, lung and spleen were collected from three Large White pigs at age of 4 month and then immediately frozen in liquid nitrogen and stored at -80°C. The DNA was extracted using the phenol–chloroform (Invitrogen, USA). Total RNA was extracted using the TRIzol reagent (Invitrogen, USA) and was reverse transcribed to cDNA using the PrimeScript^TM^ RT Reagent Kit (Takara, Japan).

Mouse myoblast cell line C2C12, mouse embryonic fibroblast-adipose like cell line 3T3-L1 and porcine kidney cell line PK15 were purchased from the Institutes for Biological Sciences Cell Resource Center, Chinese Academy of Sciences, Shanghai, China. Porcine intramuscular fat (IMF) cells were obtained according to the description in previous report [32].

### Tissue expression profile analysis by RT-PCR

Using RT-PCR, ten tissues including heart, liver, spleen, lung, kidney, stomach, brain, longissimus dorsi, fat and small intestine from three 4-month-old Large White pigs were used to analyze the expression profile of *Prdx6* gene. RT-PCR was performed using the CFX96 System (Bio-Rad, USA) in a total volume of 20 μl containing SusPr6DL-F/R or β-actin-F/R primers (Table 1) and SYBR-GreenⅡ mix (Takara, Japan). The gene expression results were normalized to the basal level of β-actin. The Ct (2^-△△Ct^) method was used to analyze the relative gene expression data.

**Table 1:** Primer/siRNA sequences for the experiments.

Name	sequences (5′-3′)	Size (bp)	Location
D1	**AGATCT**GATTGGCACGCATACAC	1510	(-1442∼+68)
D2	**AGATCT**ACATAGTCGGCAACGC	1247	(-1179∼+68)
D3	**AGATCT**GTGGCACATAACCTTACTTT	1041	(-973∼+68)
D4	**AGATCT**TGGGTCTTGCCACATT	643	(-575∼+68)
D5	**AGATCT**GCCCAGGGCTGTTCTGTC	328	(-260∼+68)
D5.1	**AGATCT**CGCCAAGGCCTGGCC	215	(-147∼+68)
D5.2	**AGATCT**CGGCCCCGCCCATCTT	181	(-113∼+68)
D6	**AGATCT**CTTGGCTGTGCTTTGGTC	168	(-100∼+68)
D7	**AGATCT**CAACACCTACAAGGCTAGAAC	84	(-16∼+68)
D8	**AGATCT**GTTAGCTGTCGCTGCTGG	54	(+15∼+68)
R	**CCATGG**CATGGCGGCAGCAGTGACGC		
hsf1-F	GG**GGTACC**ATGGATCTGCCCGTGGGC	1557	
hsf1-R	GC**TCTAGA**CTAGGAGACAGTGGGGTC		
C/EBPβ-DL-F	AAGCACAGCGACGAGTACAA	157	
C/EBPβ-DL-R	ACAGCTGCTCCACCTTCTTC		
MyoD-DL-F	AGACCACTAACGCCGACCGC	286	
MyoD-DL-R	GCGTCTGAGTCACCGCTGTAGT		
CREB-DL-F	CATGGAATCTGGAGCAGACAA	109	
CREB-DL-R	CTGGGCTAATGTGGCAATCT		
SP1-DL-F	ACATGATGACCCAGCAGGTG	280	
SP1-DL-R	TGTGAAGCGTTTCCCACAGT		
HSF1-DL-F	GGAAGCAAGAGAGCATGGAT	123	
HSF1-DL-R	TGAGCTTGTTGACGACTTTCT		
SusPr6DL-F	CAGAATTTGCCAAGAGGAAT	284	
SusPr6DL-R	GTGGTAGCTGGGTAGAGGAT		
β-actin-F	CCAGGTCATCACCATCGG	158	
β-actin-R	CCGTGTTGGCGTAGAGGT		
M-C/EBPβ-F	CAACGCAAGGGTTATTG***A***AT***C***AACTACTATCATCATC		
M-C/EBPβ-R	GATGATGATAGTAGTT***G***AT***T***CAATAACCCTTGCGTTG		
M-MyoD-F	GTGGAAACGGATGCA***TA***TGACAATGGTAACC		
M-MyoD-R	GGTTACCATTGTCA***TA***TGCATCCGTTTCCAC		
siC/EBPβ-F	CCCUGAGUAAUCACUUAAATT		
siC/EBPβ-R	UUUAAGUGAUUACUCAGGGTT		
siCREB-F	GGACCUUUACUGCCACAAATT		
siCREB-R	UUUGUGGCAGUAAAGGUCCTT		
Chip-CREB-1F	CCAAGCACTTACTGACATAG	125	
Chip-CREB-1R	CTAGCCATCCTGATTTCTG		
Chip-CREB-2F	CTATTGCGTTCCGCTTCCTT	109	
Chip-CREB-2R	CTCTAACCTGCCAGGATCC		
Chip-C/EBPβ-F	CATAGTCGGCAACGCAAG	159	
Chip-C/EBPβ-R	GCCTCTGGGTTAATGTAG		

The transcription start site (TSS) determined by 5′ RACE was defined as +1. The bold font sequences were the restriction endonuclease digested site (**AGATCT**: *Bgl*Ⅱ; **CCATGG**: *Nco*I; **GGTACC**: *Kpn*I; **TCTAGA**: *Xba*I). The mutation sites were shown by the nucleotides in bold and italic formats.

### 5′ RACE and Computational analysis

5′ RACE was performed using a Smarter™ RACE cDNA Amplification Kit (Clontech, USA), according to the manufacturer’s user manual. Total RNA of liver tissue from the same 4 month Large White pig was chosen as template for synthesis of Prdx6 cDNA. The gene specific primer (GSP) was synthesized by Sangon (China) as following sequence, GSP: 5′-GACCATCACGCTGTCTCCATTCTTCC-3′. The PCR products were purified and cloned into pMD-18T vector (Takara) and transformed into DH5α bacteria. Eighteen positive colonies were selected and commercially sequenced by Shanghai Sangon.

Transcription factor binding sites were predicted using the TFsearch (http://www.cbrc.jp/research/db/TFSEARCH.html) and TESS (http://www.cbil.upenn.edu/tess). Multiple sequence alignment was performed using the ClustalW2.0 (http://www.clustal.org).

### Plasmid construction

Ten *Prdx6* promoter deletion fragments (D1-D8) were amplified with primers listed in Table 1. PCR products were cloned into pMD-18T vector (Takara). The recombinant plasmids were then digested with *Nco*I and *Bgl*Ⅱ (Thermo, USA), and then subcloned into luciferase reporter vector pGL3-Basic (Promega, USA) respectively.

The C/EBPβ mutated vector and MyoD mutated vector were constructed by the PCR-based site-directed mutagenesis using the KOD-Plus-Neo(Toyobo, Japan) and *Dpn*Ⅰ(Thermo, USA), following the manufacturer’s protocol. The pGL3-D2 vectors containing the C/EBPβ and MyoD binding sites were chosen as template. The mutation primers M-C/EBPβ-F/R and M-MyoD-F/R were listed in Table 1. The HSF mutated D7 and Sp1 mutated D5 fragments were directly synthetized at Sangon Biotech (China) and then cloned into luciferase reporter vector pGL3-Basic as described before.

The HSF1 eukaryotic expression vector (pcDNA3.1-HSF1) was constructed. Using the cDNA (from pig liver tissue) as template, the coding sequence of *HSF1* gene (NCBI No: NM_001243819.1) was amplified with hsf1-F/R primers (Table 1). The PCR products were digested with *Kpn*I and *Xba*I (Thermo), and cloned into pcDNA3.1 vector (Promega) using T4 DNA Ligase (Takara). Other eukaryotic expression vectors (pcDNA3.1-C/EBPβ, CREB, MyoD and Sp1) used in over-expression experiment were from our laboratory.

### Cell culture, transfection and luciferase assay

The C2C12, 3T3-L1 and PK15 cells were cultured in DMEM medium (Gibco, USA) supplemented with 10% fetal bovine serum (Gibco, Australia) at 37°C and 5% CO_2_. Cells were seeded into 24-well plate and after 24 h incubation, each well was transfected with 1.0 μg of DNA constructs and 50 ng of Renilla luciferase reporter plasmid (pRL-TK, Promega) using 2 μl lipofectamine 2000 (Invitrogen, USA). For the co-transfection groups, each well was transfected with 0.8 μg of *Prdx6* promoter constructs, 0.5 μg of over-expression vector and 50 ng pRL-TK using 2 μl lipofectamine 2000. At 24–36 h after transfection, cells were lysed and assayed for the luciferase activities using dual-luciferase reporter assay system (Promega). The luciferase activity was measured using PerkinElmer 2030 Multilabel Reader (PerkinElmer) and normalized on the basis of Renilla activity.

### Transfection of plasmid DNA or siRNA oligonucleotides

PK15 cells were seeded into 6-well plate and cultured at 37°C and 5% CO_2_. After 24 h incubation, cells of each well were transfected with 4μg of over-expression vector or pcDNA3.1 empty vector using 9 μl lipofectamine 2000. C2C12 cells were seeded into 6-well plate and cultured at 37°C and 5% CO_2_. When reaching 70% confluence, cells of each well were transfected with 100 pmol of siRNA using 9 μl lipofectamine 2000. The C/EBPβ and CREB siRNAs (Table 1) were purchased from genepharma (China). After transfection for 36 h, cells were collected; RNA and proteins were extracted for quantitative RT-PCR and Western Blot detection respectively. RNAs were extracted using HP Total RNA Kit (OMEGA, USA) according to the manufacturer’s instructions. The reverse transcription and RT-PCR were performed as described before. Primers used in RT-PCR were listed in Table 1.

### Western blot

After transfection for 36 h, Cell lysates were prepared in ice-cold RIPA lysis buffer according to manufacturer’s instruction (Beyotime, China). Equal amounts of protein samples were loaded into a 10% SDS-PAGE and transferred to PVDF membrane (Millipore Billerica, MA, USA). After 1.5 h blockading within 5% nonfat dried milk, membranes were incubated overnight with primary antibodies at 4°C. After washing for 3 times, membranes were incubated with secondary antibodies for 1.5 h at room temperature. The results were visualized using the ECL Western Blotting Detection System (Bio-Rad, USA). Primary antibodies contain anti-Prdx6 (Santa Cruz, SC-134478; 1:500 dilution), anti-C/EBPβ (Santa Cruz, SC-150X; 1:500 dilution), anti-CREB (Cell Signaling Technology, #4820; 1:1000 dilution), anti-HSF1(Cell Signaling Technology, #4356P; 1:1000 dilution) and β-actin antibody (Boster, China, BM0627; 1:1000 dilution). Goat anti-rabbit IgG-HRP and goat anti-mouse IgG-HRP secondary antibodies (Boster, China; 1:3000 dilution) were used in this study.

### Electrophoretic mobility shift assay (EMSA)

Nuclear extracts were prepared from C2C12 cells or porcine intramuscular fat (IMF) cells with the Nuclear Extraction Kit (Active Motif, CA, USA). EMSA was performed according to the manufacturer’s instruction of Chemiluminescent EMSA Kit (Beyotime). The biotin-labeled double-stranded oligonucleotides (Sangon) containing predicted CREB or C/EBPβ binding sites were incubated with 10 μg nuclear extracts for 20 min at room temperature and were subjected to electrophoresis on 6% polyacrylamide gels. Then transferred to a nylon membrane and results were visualized using the ECL (Bio-Rad). In competition group, a 1-fold molar excess of unlabeled oligonucleotides was added to the reaction mixture prior to the addition of biotin labeled probe. For the supershift group, 2 μg C/EBPβ (Santa Cruz) or CREB (Cell Signaling Technology) antibodies was added to verify the specificity of DNA-protein interaction. The EMSAs were performed according to previous reports [33, 34].

### Chromatin immunoprecipitation (ChIP) assay

ChIP assay was carried out using the ChIP assay Kit (Beyotime) following the manufacturer’s protocol. The PK15 cells cultured in 15 cm-diameter dishes were fixed in formaldehyde for 20 min at room temperature and neutralized with glycine for 5 min. After washing with cold PBS, scraped and harvested cell lysates were sonicated to produce chromatin fragments about 200–750 bp in size. An equal amount of chromatin was immunoprecipitated overnight at 4°C with 4 μg of the following antibodies: anti-CREB, anti-C/EBPβ and Normal Rabbit IgG, and added the ProteinA+G Agarose beads (Beyotime) for further 5 h incubation. Then the beads were washed, and the bound chromatin was eluted in ChIP Elution Buffer. The DNA was treated with proteinase K for 4 h at 45°C. After purified, PCR was carried out using 3 μl DNA sample with primers specific to the *Prdx6* promoter (Table 1). The first binding region of CREB was amplified by PCR as the following program: 30 cycles at 94°C for 30 s, 50°C for 30 s, 72°C for 15 s. The second binding region of CREB was amplified by PCR as the following program: 30 cycles at 94°C for 30 s, 55°C for 30 s, 72°C for 15 s. The binding region of C/EBPβ was amplified by PCR as the following program: 35 cycles at 94°C for 30 s, 50°C for 30 s, 72°C for 15 s. The PCR products were visualized on a 2.0% agarose gel. Total chromatin was used as input. The immunoprecipitate from Normal Rabbit IgG group was used as negative control.

### Immunoprecipitation

PK15 cells cultured in 10 cm-diameter dishes were collected and lysed in nondenaturing lysis buffer (Sangon, Shanghai, China) supplemented with protease and phosphatase inhibitor cocktails. Well equal mass of lysate was incubated overnight with 2 μg either anti-Prdx6 or anti-IgG together with 25 μl Protein A+G Agarose beads. After centrifuge the beads were washed with 1ml lysates buffer for three or four times. Then the beads were added with 15 μl 2×SDS loading buffer and boiled for 10 minutes, then were loaded onto an SDS-PAGE gel for western blot analysis.

### Statistical analysis

Statistical analysis was performed using Student’s *t* test. Data are expressed as mean ± S.D., and statistical significance was considered as *p* < 0.05. **P<0.01; *P<0.05; NS, not significant.

## Results

### Identification of transcription start site of porcine *Prdx6* gene

**Fig 1:**
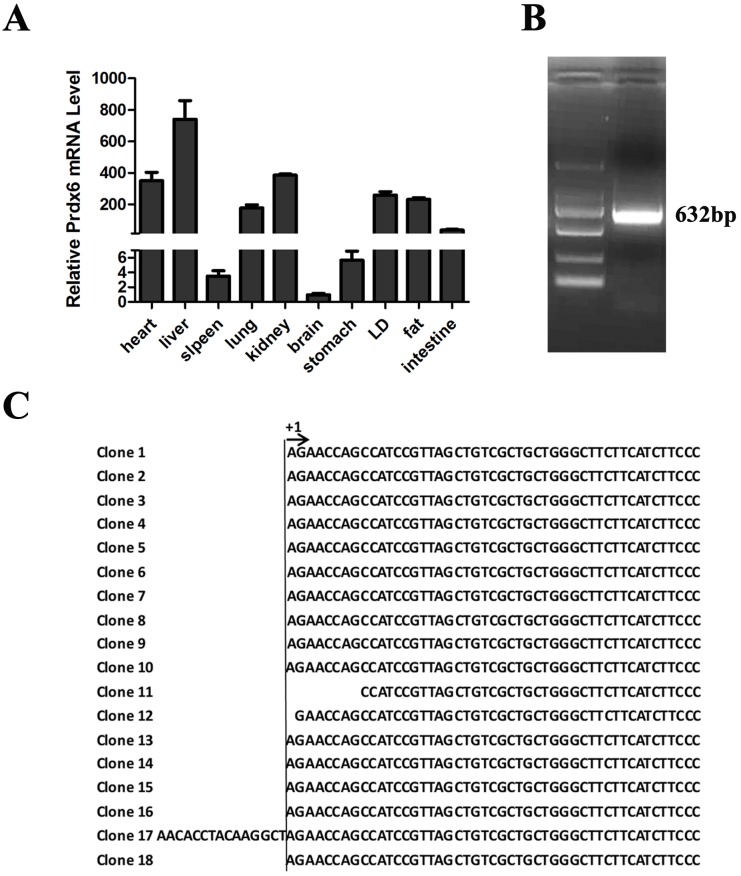
Identification of *Prdx6* TSS using 5′ RACE. (A) The *Prdx6* mRNA expression profile was analyzed by RT-PCR. (B) 5′ RACE PCR product of 632bp was visualized in 1.5% agarose gel. (C) Eighteen sequencing results of *Prdx6* 5′ RACE clones are listed and the major TSS adenine was defined as +1.

To better understand the transcription regulation of porcine *Prdx6* gene, we determined the exact TSS of *Prdx6* by 5′ RACE. Firstly, the tissue expression profile of *Prdx6* was analyzed by real time-PCR. The *Prdx6* was expressed in almost all the tissues, especially with the highest expression in liver tissue; the kidney, heart, longissimus dorsi (LD), fat and lung tissues also showed higher expression level; while a very low expression level was detected in brain tissue (Fig 1A), which was consistent with the previous report [12]. Then we selected liver tissue as the sample for 5′ RACE to determine the TSS of *Prdx6*. And the PCR products of 632bp were obtained (Fig 1B). After cloning and sequencing, the adenine located at 65bp before the first exon was identified as the major TSS as it appears 15 times in the total 18 clone sequencing results (Fig 1C), while some RACE clones (clone 11, 12 and 17) showed slightly different TSS which were located at the 57bp, 64bp and 80bp before the first exon, respectively. This is a common feature observed in promoter of less TATA-box [35], and *Prdx6* gene belongs to the TATA-less promoter. We defined the major TSS as +1 for later illustration.

### Identification of crucial transcriptional factors controlling *Prdx6* gene expression

**Fig 2:**
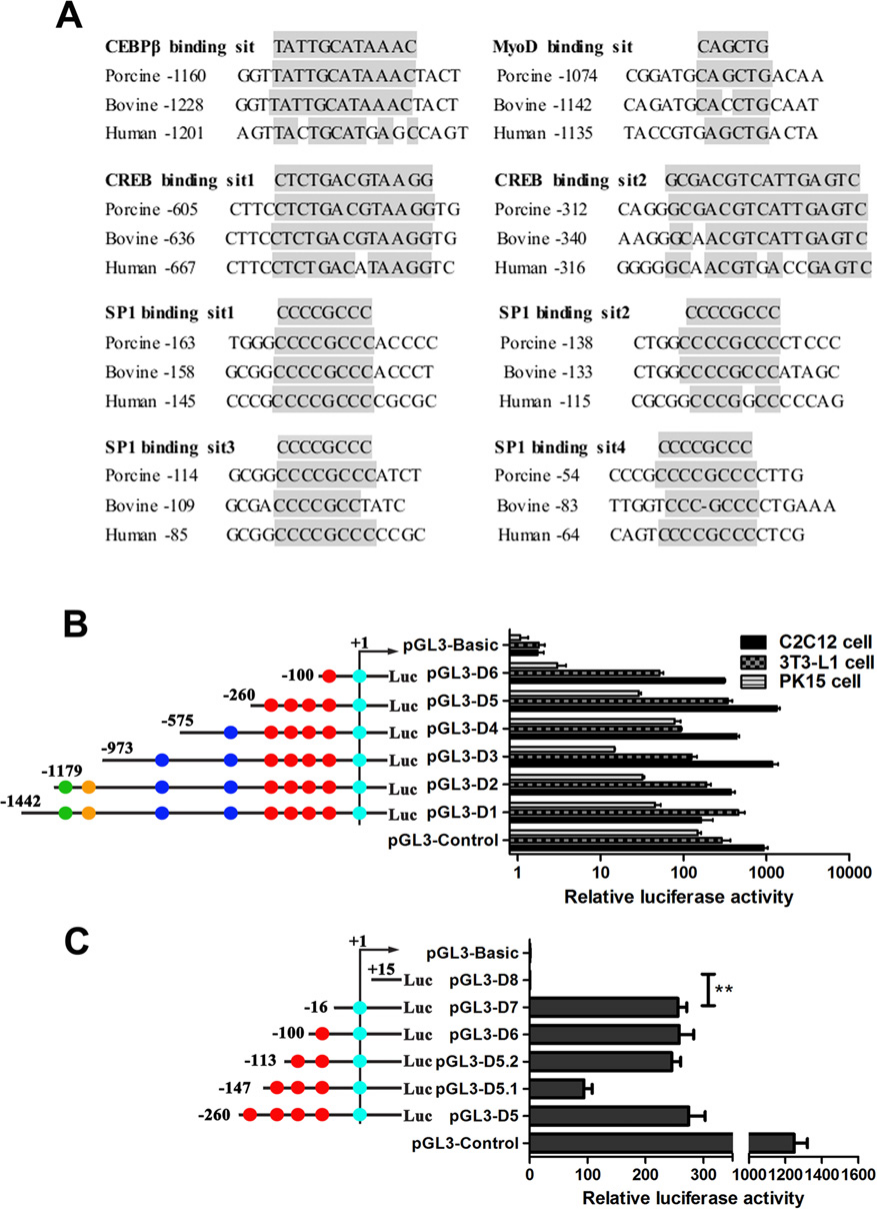
The porcine Prdx6 gene promoter was analyzed by computational analyses together with luciferase reporter system. (A) Conserved sequences of transcription factor binding sites of *Prdx6* promoter. The aligned region is conserved among pig, human and bovine. (B) Six promoter constructs were transfected into C2C12, 3T3-L1 and PK15 cell lines, and assayed for luciferase activity. The pGL3-Control and pGL3-Basic were the positive and negative control respectively. Green, orange, blue, red and bright blue dots on the left panel represented the C**/**EBPβ, MyoD, CREB, Sp1 and HSF binding sites predicted on TFsearch or TESS websites, respectively. Data are expressed as means ± SD of three replicates. (C) Four further deletions (D5.1, D5.2, D7, and D8) were transfected into C2C12 cells and assayed for luciferase activity.

Using the TFSEARCH and TESS website analysis, a series of transcription factor binding sites related to muscle or fat development were found in the porcine *Prdx6* promoter region, such as C**/**EBPβ (-1156/-1146), MyoD (-1068/-1063), CREB (-601/-588; -308/-293). Besides, four GC-boxes (Sp1-response element) were found at the proximal region (-161/-153; -136/-128; -112/-104; -52/-44). By alignment of multiple sequences, higher similarities of those factor binding sites were found among pig, bovine and human (Fig 2A). This suggested that *Prdx6* gene might be regulated by multiply transcription factors.

To determine the crucial promoter motifs, six promoter deletions (D1-D6) were linked to the pGL3-basic vector, and their luciferase activity was analyzed in PK15, C2C12 and 3T3-L1 cell lines (Fig 2B). The promoter activity was with the highest expression in C2C12 cells and the lowest expression in PK15 cells, which may reflect the difference of transfection efficiency in different cell lines and the transcriptional regulation in different species. Compared with the basal level of pGL3-basic, every deletion showed significant promoter activity in each cell line. Generally, D5 deletion had the highest promoter activity; the shortest D6 deletion had the lowest one; while the promoter activities of other deletions were between them. This experiment further indicated that the regulation of Prdx6 gene is complex, and it might be regulated by multiple transcription elements. For example, the fragment from -575bp to -973bp of D3 deletion may contain crucial *cis* elements which are important for *Prdx6* gene expression in C2C12 cells since the luciferase activity of D3 deletion is much higher than that of D4 deletion in C2C12 cells. The D5 and D6 deletions may also contain crucial *cis* elements which are vital for *Prdx6* transcriptional initiation since the shortest two deletions have significant luciferase activity compared with negative control. We then constructed four deletion fragments (D5.1, D5.2, D7 and D8) to further analyze the transcriptional activity of *Prdx6* promoter. The results showed that the minimal transcription activity region was located at -16bp~+15bp containing one HSF binding site (Fig 2C).

### C/EBPβ and CREB increase *Prdx6* gene expression

**Fig 3:**
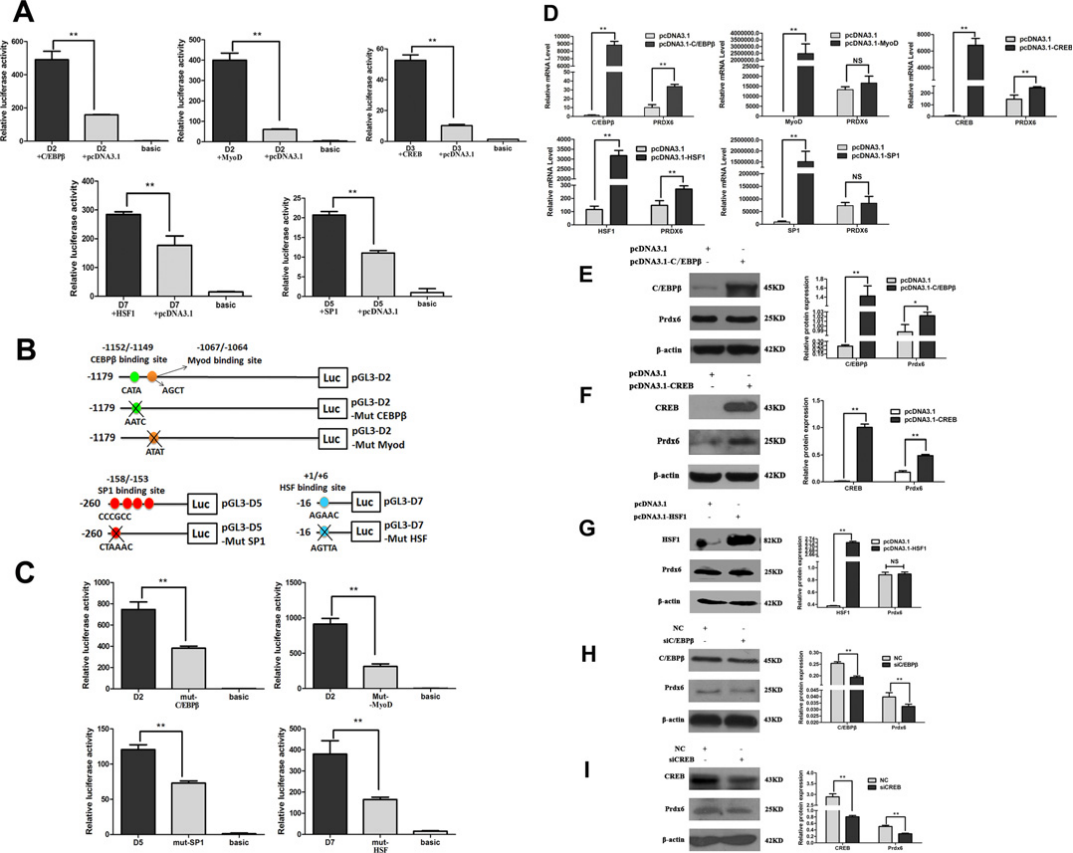
C/EBPβ and CREB could up-regulate the *Prdx6* expression. (A) The Prdx6 deletions were co-transfected with relative overexpressed vector or pcDNA3.1 empty vector. The promoter activity was measured and the results were expressed as means± SD of three replicates. (B) Schematic structure of site-direct mutants of *Prdx6* promoter linked to pGL3-Basic vector. (C) Wild-types and mutants of the *Prdx6* deletions were transfected into C2C12 cells, and luciferase activity was detected and the results were expressed as means± SD of three replicates. (D) The *Prdx6* mRNA expression level was detected by RT-PCR after overexpression of each transcription factor into PK15 cells, data were showed as means± SD of three replicates. (E-G) The transfection efficiency of C**/**EBPβ, CREB or HSF1 overexpression vector and their effect on target Prdx6 protein expression level were determined by western blot. (H-I) The interference efficiency of Knockdown of C**/**EBPβ or CREB and their effect on target Prdx6 protein expression level were determined by western blot. Quantification results of western blot represented by ratio of C**/**EBPβ, CREB, HSF1 or Prdx6 to β-actin protein expression level (Image J software).

Co-transfection experiments were performed to identify the transcription factor regulating the *Prdx6* gene transcription. As shown in Fig 3A, co-transfection of Prdx6 deletion fragments with over-expression vectors of each transcription factor could remarkably increase the luciferase activities compared with the co-transfection with pcDNA3.1 empty vector. Then we mutated the C/EBPβ, MyoD, Sp1 and HSF binding sites in the Prdx6 deletion plasmids (Fig 3B), and found sharply decreased luciferase activity of the mutated group compared with the wild-type group (Fig 3C). To determine which factor can indeed affect the *Prdx6* transcription at mRNA level, five transcription factors were transfected into PK15 cells. The RT-PCR results revealed that C**/**EBPβ, CREB and HSF could improve *Prdx6* transcription level, while MyoD and Sp1 had no effects (Fig 3D). Prdx6 protein level was also increased after transient transfection with pcDNA3.1-C/EBPβ or pcDNA3.1-CREB (Fig 3E and 3F), but nearly no change after transfected with the pcDNA3.1-HSF1 vector (Fig 3G). Inhibition of C/EBPβ or CREB by siRNA could decrease Prdx6 protein level (Fig 3H and 3I). Those data indicated that C/EBPβ and CREB increase *Prdx6* expression both at mRNA and protein level.

### CREB could bind to the first CRE site of *Prdx6* promoter *in vivo* and *in vitro*

**Fig 4:**
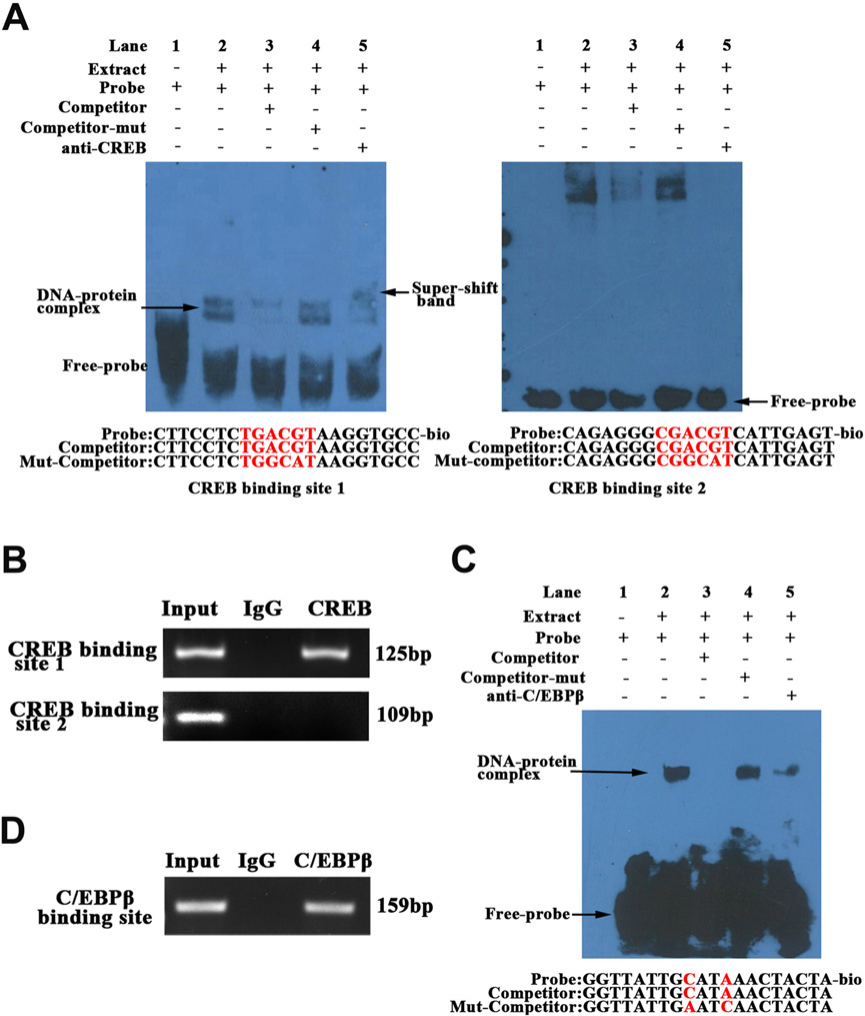
C/EBPβ and CREB bind to *Prdx6* promoter region *in vitro* and *in vivo*. (A) Binding of CREB with *Prdx6* promoter was detected by EMSA with C2C12 cell nuclear extracts. Probes of CREB binding sites were labeled with biotin and the mutated nucleotides were depicted in red color. Lane1 was a blank control that did not add nuclear extracts; Lane2 was experimental group. For Lane3 and 4, a 1-fold excess of unlabeled or mutant probe was added to the reaction complex. The Lane5 was added 2μg anti-CREB antibody in the complex. The DNA-protein complex and the super-shift bands were indicated by arrows (B) ChIP assay of CREB binding to the *Prdx6* promoter in PK15 cells. (C) The interaction of C**/**EBPβ with *Prdx6* promoter was detected by EMSA with the porcine IMF cell nuclear extracts. The Lane1-5 experimental groups are similar to figure 4A. (D) Binding of C**/**EBPβ on the *Prdx6* promoter was determined by ChIP assay.

The EMSAs were performed with nuclear extracts from C2C12 cells to determine whether CREB bind to Prdx6 promoter (Fig 4A). For the first CREB binding site (-601/-588), the DNA-protein complex were obviously present in the biotin-labeled probe (Lane2) and mutation-competitor (Lane4) groups, whereas no complexes were observed in the absence of nuclear extracts (Lane1). The cold-competitor group (Lane3) displayed decreased intensity of the complex. The specific anti-CREB antibody was added to the mixture of Lane5 and caused the weakness of DNA-protein complex and the appearance of super-shift band. For the second CREB binding site (-308/-293), no specific DNA-protein complex was detected (Fig 4A right). These results indicated that CREB could bind to the first CREB binding site of *Prdx6* promoter.

ChIP assay was preformed to confirm the binding of CREB to the *Prdx6* promoter *in vivo*. Chromatin fragments from lysates of PK15 cells were precipitated with a CREB antibody or control IgG (Fig 4B). The immunoprecipitates were analyzed by PCR with primers specific to the *Prdx6* promoter harboring the CREB binding site. The results also revealed that only the first CRE site of *Prdx6* promoter could bind to CREB *in vivo*. These results suggested that the CREB promotes the expression of *Prdx6* gene through binding to the first rather than the second CRE site. This also explained why the luciferase activity of D3 deletion (with two CRE site) was significantly higher than that of D4 deletion (with only the second CRE site) detected in C2C12 cells (P = 0.003) (Fig 2B). No significant difference of Luciferase activity between D3 and D4 detected in 3T3-L1 cells (P = 0.052) may due to the relatively lower expression of CREB in this cell line. In PK15 cells, a completely opposite result was obtained, which may result from the different transcriptional regulation mechanism existed in this cell line.

### C/EBPβ could bind to the *Prdx6* promoter *in vivo* and *in vitro*

By EMSA and ChIP assays, we detected the interaction of C/EBPβ with Prdx6 promoter *in vitro* and *in vivo* (Fig 4C and 4D). The specific DNA-protein complex was found in biotin-labeled probe group (Lane2), and was attenuated after addition of cold-competitor probe (Lane3), while the mutated competitor probe failed to abolish complex formation (Lane4). The specific complex did not appear without nuclear extracts in Lane1. Although there is no super-shift band appearance in lane5, the complex band was also diminished after addition of C/EBPβ antibody (Fig 4C). Therefore, we infer that the super-shift band may exist but were not exhibited due to the less sensitivity. ChIP results showed that the specific DNA fragments containing the C/EBPβ binding site of *Prdx6* promoter could be amplified in the anti-C/EBPβ group immunoprecipitates rather than the IgG group (Fig 4D). The results indicated that C/EBPβ could bind to the *Prdx6* promoter and increase its expression. In 3T3-L1 and PK15 cells, the luciferase activities of D1 and D2 deletions (containing C/EBPβ binding site) were significantly higher than those of D3 deletion (no C/EBPβ binding site) (Fig 2B), which was consistent with our EMSA and ChIP results. In C2C12 cells, the opposite results were found, because the C/EBPβ was higher expressed in fat cells like 3T3-L1 rather than C2C12 cells.

### Prdx6 could interact with C/EBPβ *in vivo*

**Fig 5:**
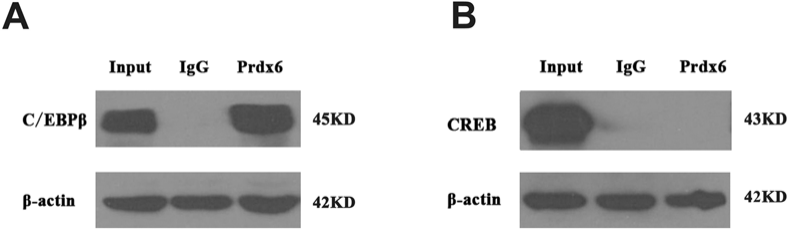
Prdx6 could interact with C/EBPβ *in vivo*. Immunoprecipitation was performed in PK15 cells using anti-Prdx6 and anti-IgG antibodies. Total Protein was used as input. The protein from Normal rabbit IgG group was used as negative control. (A) Detection of C/EBPβ protein in immunoprecipitation. (B) Detection of CREB protein in immunoprecipitation.

Although the transcription regulation mechanism of porcine *Prdx6* gene by CREB and C/EBPβ have been illustrated, the interaction of Prdx6 protein with CREB and C/EBPβ proteins were still not clear *in vivo*. To address this, the immunoprecipitation was performed in PK15 cells. Proteins from the PK15 cells were collected and immunoprecipitated by Prdx6 antibody or IgG antibody. The results showed that compared with IgG group, C/EBPβ protein was detected in the immunoprecipitates of anti-Prdx6 group (Fig 5A), whereas CREB could not be detected (Fig 5B), demonstrating that Prdx6 could interact with C/EBPβ and might participate in its biological function.

## Discussion

In this study we determined the core promoter motifs and key regulators controlling the transcription of porcine *Prdx6* gene. By 5′ RACE, we analyzed the *Prdx6* transcription start site for the first time and found that it is different from the computational prediction. The TSS of *Prdx6* is an adenine located at 65bp before the first exon. Bioinformatic analysis and experiments revealed some important *cis* element binding sites including C**/**EBPβ, MyoD, CREB, Sp1, and HSF. Our data indicated that only C**/**EBPβ and CREB could up-regulated the *Prdx6* expression.

The CCAAT/enhancer-binding proteinβ (C/EBPβ) is a basic leucine zipper DNA-binding factor and could recognize a consensus DNA sequence (TTNNGNAAT) in target gene [36]. In present study, one C/EBPβ binding sites (TTGCATAAACT) was identified in the porcine *Prdx6* promoter, which was located at -1156/-1146 bp. Co-transfection of *Prdx6* promoter deletion with a construct over-expressing C/EBPβ significantly increased *Prdx6* promoter activity (about 3-fold, P<0.01, Fig 3A). After mutated the C/EBPβ binding site of *Prdx6* promoter, the luciferase activity was significantly lower than that of wild type vector (Fig 3C). Over-expression experiment results showed the expression of the *Prdx6* gene could be up-regulated by C/EBPβ (Fig 3D and 3E). Many studies proved that C/EBPβ is an important early regulator of adipogenesis that could modulate the key adipogenic transcription factors C/EBPα, PPARγ and SREBP1 [37, 38]. By EMSA and ChIP assays, we also found the C/EBPβ could bind to *Prdx6* promoter and promote its expression. The important function of Prdx6 is scavenging and limiting reactive oxygen species (ROS) level. Previous reports indicated that oxidative stress and the generation of ROS could regulate the adipogenesis. For example, the hypoxia-inducible factor HIF1 which can inhibit PPARγ was regulated by ROS [39]. ROS have been investigated to facilitate adipogenesis by accelerating mitotic clonal expansion during 3T3-L1 cell differentiation [40]. So it is possible that the C/EBPβ affects adipogenesis through promoting the expression of *Prdx6* and thereby leads to the down-regulation of ROS levels. Besides, C/EBPβ could interact with Prdx6 protein (Fig 5A), suggesting the possibility that Prdx6 might participate in the biological function of C/EBPβ in adipogenesis. However, this possibility still needs more research.

The cAMP response element binding protein (CREB) is a cellular transcription factor that recognizes and binds to the cAMP response element (CRE, TGACGTCA) of target gene promoter [41]. By regulating the expression of its target genes CREB affect several physiological process like cell proliferation, apoptosis, brain circuits [42], and is also involved in responses to oxidative stress [43]. The thioredoxin and redox factor 1 were regulated by CREB under certain stimuli [44]. Here we found that the porcine Prdx6 is also regulated by CREB. *Prdx6* promoter region contains two CREB binding sites (-601/-588; -308/-293), and only the first one (-601/-588) could bind with CREB and affect the *Prdx6* promoter activity by EMSA, ChIP and over-expression experiments (Fig 3F; Fig 4A and 4B). Previous studies have proved that CREB plays a vital role in muscle differentiation and regeneration. For example, Zuloaga et al proved that the activated CREB by insulin-like growth factor-1(IGF-1) regulates *myostatin* expression and further inhibits muscle differentiation [45]. Stewart et al found that the CREB is activated by muscle injury and promotes muscle regeneration [46]. CREB-induced up-regulation of C/EBPβ is required in infiltrating macrophages for induction of M2-speciﬁc genes expression and muscle regeneration [47]. The present study demonstrated the Prdx6 could interact with C/EBPβ, which might provide a new insight of Prdx6 function in muscle differentiation and regeneration through CREB/C/EBPβ cascade. Moreover, ROS is an important signal molecule that plays an essential role in muscle differentiation [48, 49]. Since its important role in regulating ROS level, we hypothesized that Prdx6 participate in muscle development by eliminating ROS.

The present study also analyzed several other potential regulators of porcine *Prdx6* gene, such as Sp1, MyoD and HSF. *Prdx6* promoter contains four conserved GC-boxes but no TATA-box. According to the previous study, the TATA-less promoter is likely to be regulated by GC-box and its response element Sp1 [50]. A conserved E-box (-1068/-1063, CAGCTG), the bHLH (basic helix loop helix) transcription factor MyoD binding site, was also found in the *Prdx6* promoter. Although the HSF binding site (+1/+5, AGAAC) of *Prdx6* promoter was not conserved among species, it showed remarkable effect in initiating promoter activity (Fig 2C). Co-transfection and site direct mutation assays suggesting the possibility that expression of the *Prdx6* gene might be regulated by Sp1, MyoD and HSF1. However, overexpression of Sp1 and MyoD in the PK15 cells did not change the mRNA expression level of *Prdx6* (Fig 3D). HSF1 could up-regulate the mRNA level of *Prdx6* but have no effect at the protein level of *Prdx6* (Fig 3D and 3G). The possible reason maybe two following points: (1) we just analyzed the activating effect of transcription factors to certain promoter fragment of Prdx6 gene, but ignored the complex transcriptional regulation system that may exist in *Prdx6* promoter. For *Prdx6* promoter, there is a complex of transcription sites for of the different transcription factors *in vivo* and the transcriptional regulation is dependent on this complex and not from any single individual transcriptional factor. (2) The porcine PK15 cells used in this paper were different from previous study, in which the human LEC cells were used and the Sp1 had been proved to be an activator of *Prdx6* [27]. Therefore, we infer that the regulation of *Prdx6* gene regulated by Sp1 is different among species.

**Fig 6:**
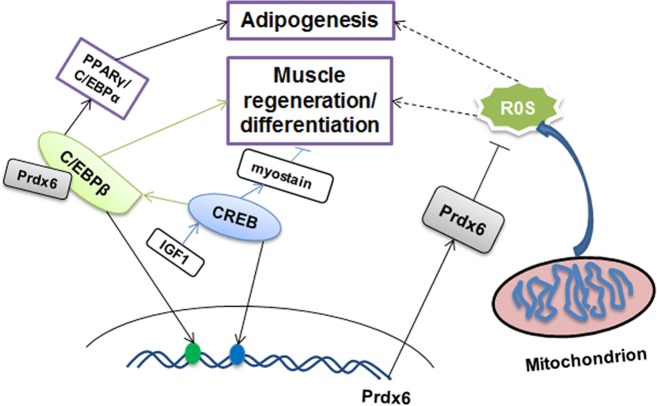
Model for mechanisms of Prdx6 participates in adipogenesis, muscle differentiation or regeneration. C/EBPβ or CREB-mediated up-regulation of Prdx6 may participate in adipogenesis, muscle differentiation or regeneration through two pathways: (1), Prdx6 inhibits adipogenesis and muscle differentiation by scavenging ROS; (2), Prdx6 interacts with C/EBPβ to participate in adipogenesis and muscle regeneration.

In conclusion, we have identified the transcription start site and important *cis* elements of porcine *Prdx6* and studied the transcription regulators affecting *Prdx6* transcription. The CREB and C/EBPβ were found for the first time to transactivate *Prdx6* gene expression by binding to the relevant promoter region. We also found the C/EBPβ could interact with Prdx6. A hypothetical model was proposed that Prdx6 might participate in adipogenesis or myogenesis by interacting with C/EBPβ or scavenging ROS level (Fig 6). The present study will help to better understand the basal transcriptional regulation mechanism and provide clues for further investigation of *Prdx6* gene function.
